# Pirfenidone-Induced Photosensitive Dermatitis: A Rare Side Effect

**DOI:** 10.7759/cureus.15200

**Published:** 2021-05-23

**Authors:** Ramsha Saleem, Sachin Vaidya

**Affiliations:** 1 Medicine, Royal Adel, Adelaide, AUS; 2 Medicine, The Queen Elizabeth Hospital, Woodville South, AUS

**Keywords:** anti-inflammatory drugs, pirfenidone, photosensitive rash, drug eruption, cutaneous immunology

## Abstract

Many classes of drugs are known to cause a photosensitive reaction, including anti-bacterial, anti-inflammatory, and nonsteroidal drugs. Pirfenidone is an anti-inflammatory drug that is used to treat idiopathic pulmonary fibrosis (IPF). We report a case of a patient who developed a photosensitive rash secondary to pirfenidone use, which resolved after discontinuing administration of the drug.

## Introduction

The word photosensitivity dermatitis is the name given to an eczematous eruption that arises in response to exposure to electromagnetic radiation. Photodermatoses are categorized to be idiopathic due to a known photosensitizer or autoimmune bullous [1]. The symptoms of a phototoxic reaction are weeping, blistering, peeling, and itching which may occur for an individual anywhere between mild and severe on the photosensitivity spectrum. The most commonly observed cause of photosensitive dermatitis is due to exposure to sunlight [2]; however, can also be drug or chemical-induced.

Drug-induced photosensitivity occurs predominantly as a phototoxic reaction that can be reversed by substituting or withdrawing the drug. Drug classes currently known to be responsible for photosensitivity are anti-bacterial, anti-inflammatory, and nonsteroidal drugs with a low (200-500 Da) molecular weight, a structure that enables resonance stabilization and high absorption of UV radiation [3]. An example of an anti-inflammatory drug leading to a phototoxic reaction is pirfenidone. This report presents a case of a successful recovery of photosensitivity after discontinuation of pirfenidone for lung fibrosis. 

This report aims to highlight the importance of maintaining clinical suspicion of drug-related reactions in patients.

## Case presentation

**Figure 1:**
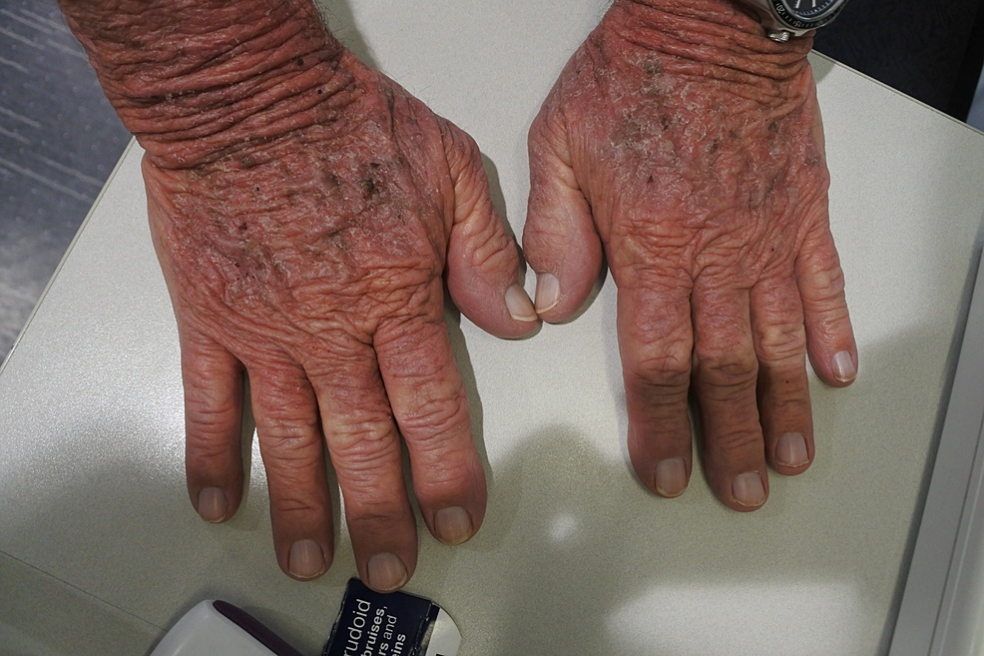
Rash on hands

**Figure 2:**
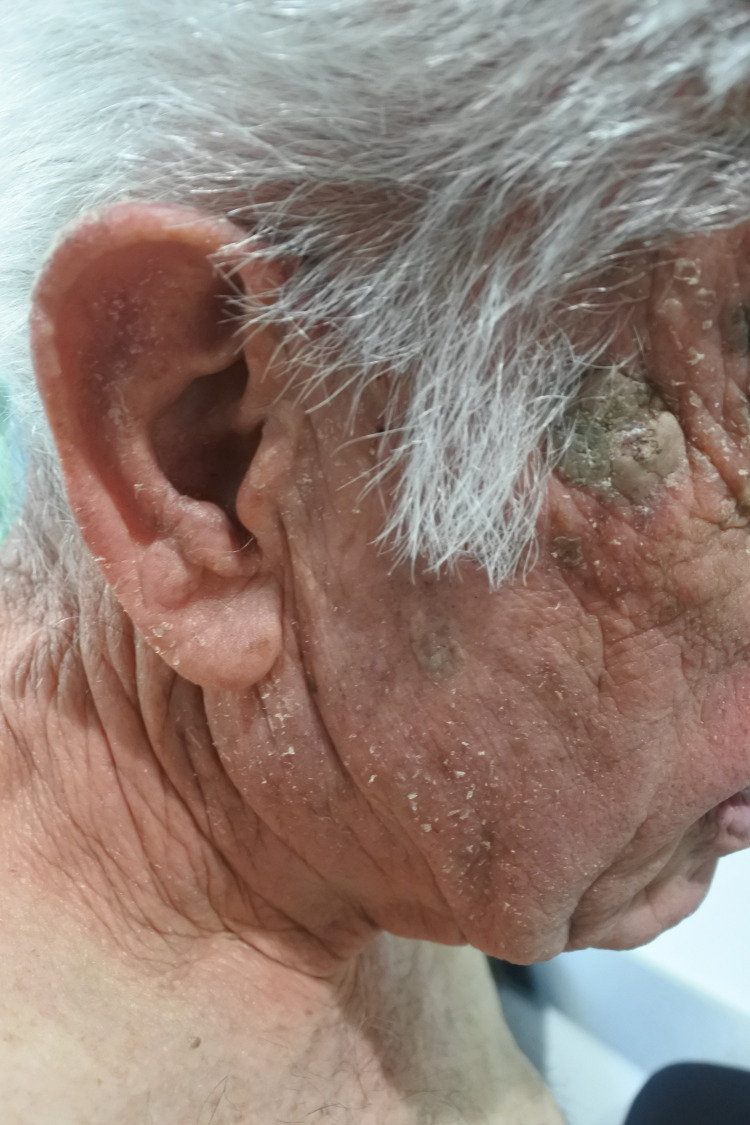
Rash on face

**Figure 3:**
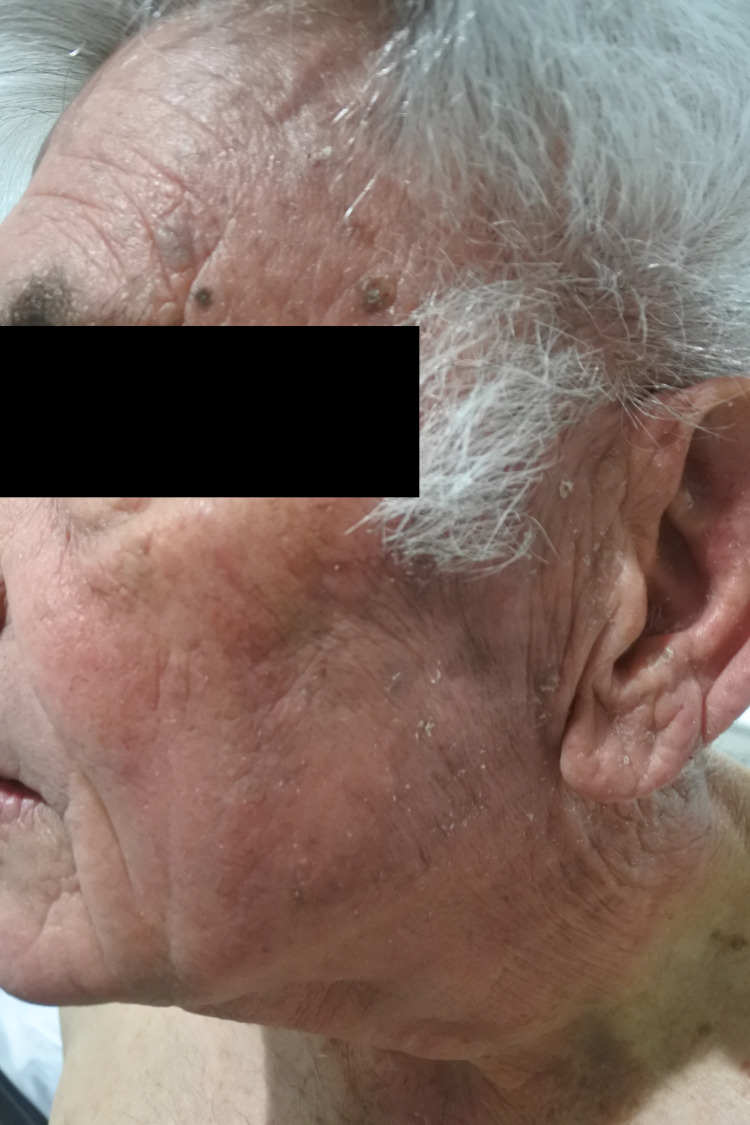
Rash on face

**Figure 4:**
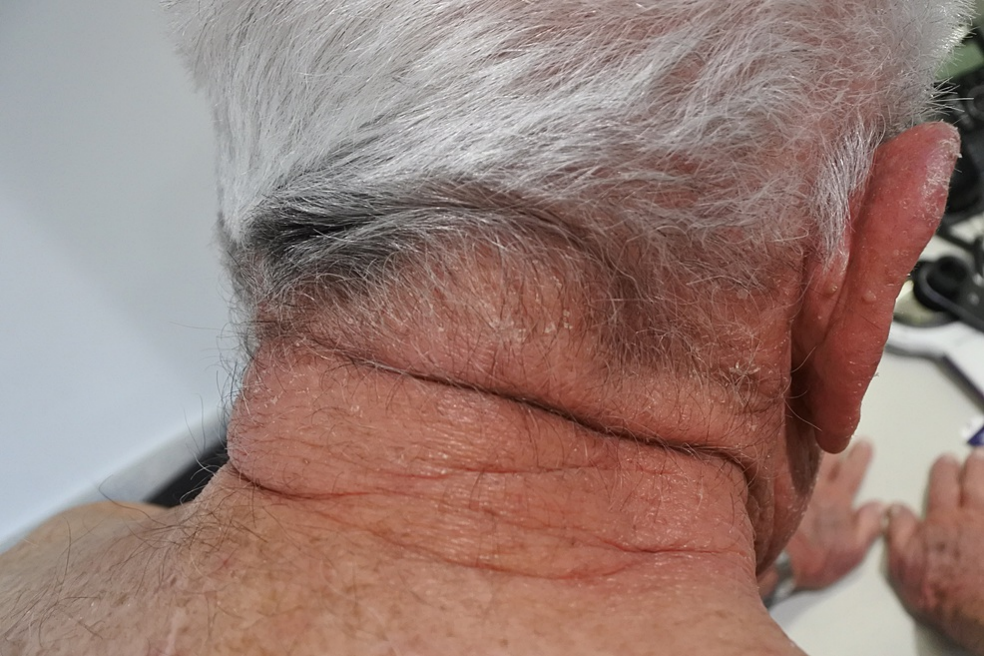
Rash on neck

A 79-year-old male patient presented to a clinic with a three-month history of an itchy, erythematous, and scaly rash on the scalp, face, neck, chest, and hands (Figures 1-4). The gentleman had a medical history of idiopathic pulmonary fibrosis (IPF) for which he was commenced on pirfenidone three months ago. With no recent changes in any other medication, a clear correlation between the commencement of pirfenidone and the emergence of dermatitis could be seen. This led to the diagnosis of a phototoxic reaction secondary to the use of pirfenidone.

Following the conclusion of causality, treatment was commenced with topical steroids to manage dermatitis. In order to manage the patient’s IPF condition alongside, consultation from a respiratory physician was sought for an alternative to pirfenidone. Consequently, the patient was switched over to nintedanib for the treatment of IPF, a drug with a similar mechanism of action but different chemical composition.

**Figure 5:**
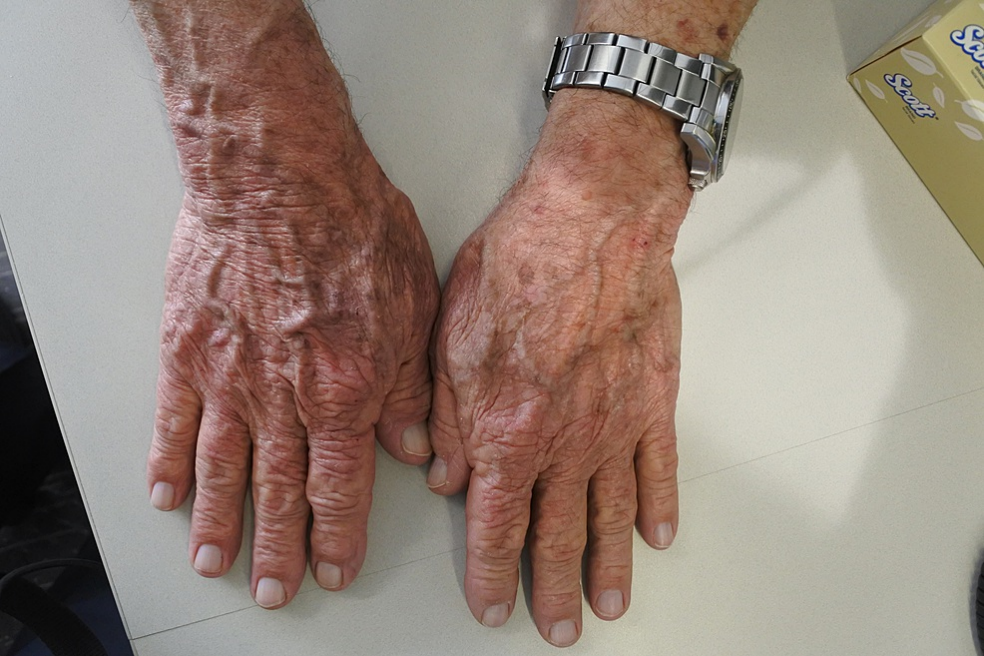
Recovered

**Figure 6:**
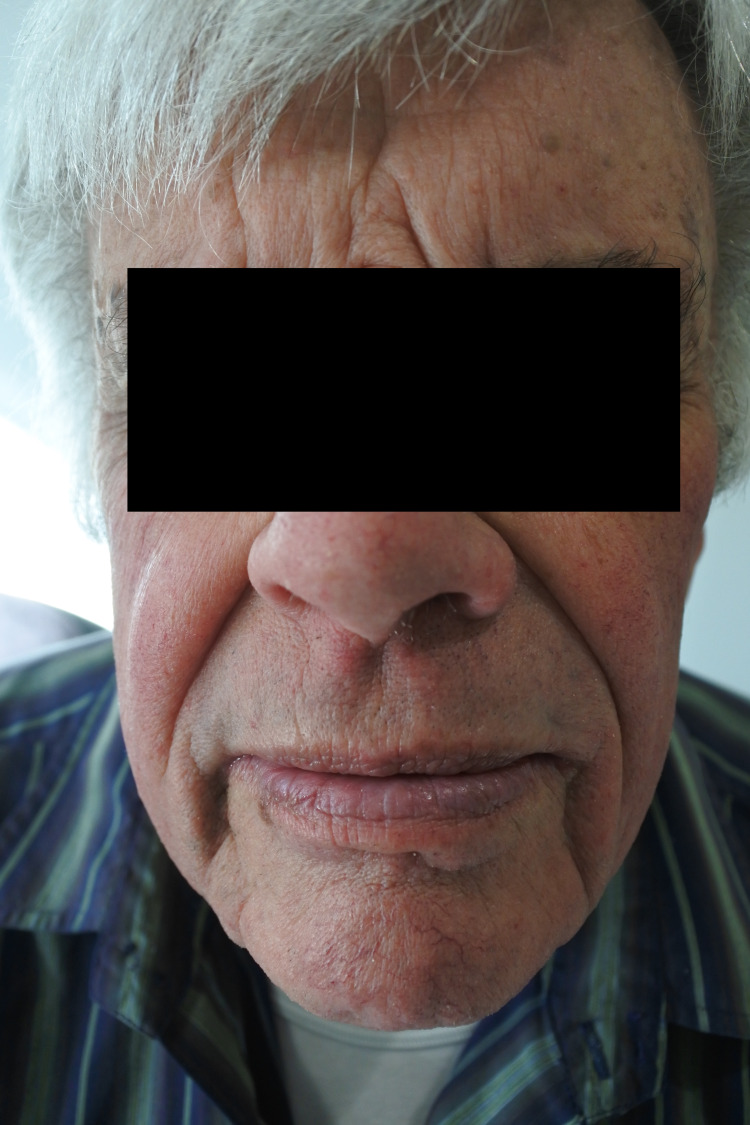
Recovered

**Figure 7:**
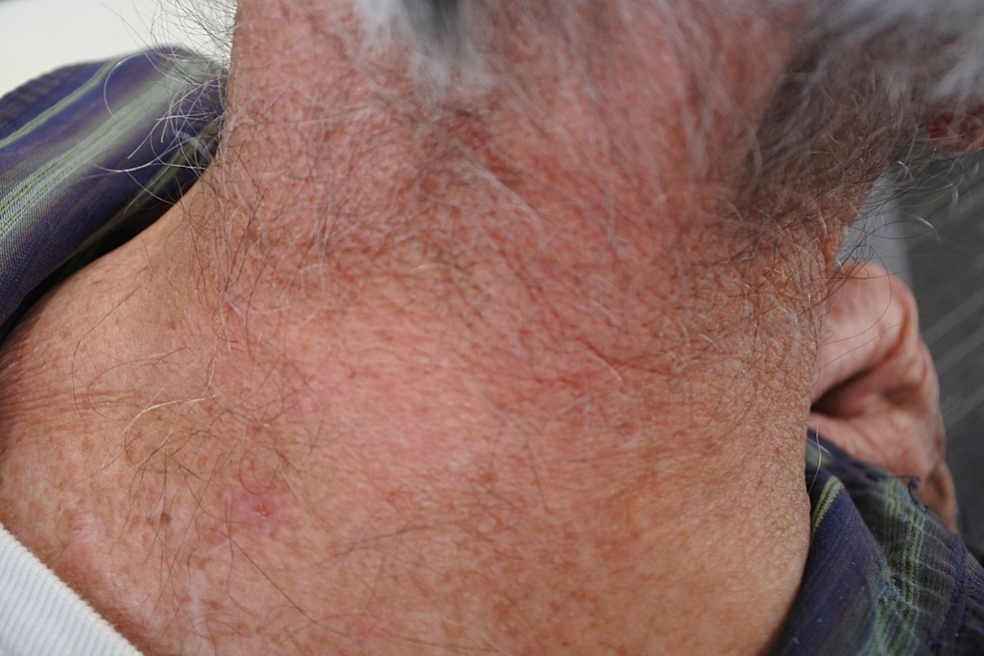
Recovered

The patient was reviewed in the clinic after six weeks and a dramatic improvement in the rash could be seen further reinforcing the importance of discontinuation of pirfenidone. At the sixth week review, only mild residual eczema on hands could be observed (Figures 5-7).

## Discussion

Released in 2008, pirfenidone is an antifibrotic and anti-inflammatory agent given to patients with declining lung function. Several studies demonstrate that the treatment reduces the decline in lung function and improves progression-free survival. Although generally well-tolerated, patients discontinue therapy due to gastrointestinal and skin-related adverse events (AEs) [4]. Reports of photosensitivity with the administration of pirfenidone vs placebo were 12% vs 2% [5]. In another study, photosensitivity reactions were reported in 24% of patients receiving high doses of the drug [4] while according to a study by Kuskabe et al. (2018) the incidence of photosensitivity post pirfenidone was 51.7% in its clinical trial stage [6]. Photosensitivity as a reaction to pirfenidone remains one of the major reasons for the discontinuation or decreased dosage of the drug.

The reported cases with serious photosensitizing drug eruptions caused by pirfenidone are due to its phototoxic effect and its ability to absorb UVA and UVB. The absorption of UV radiation by pirfenidone present in the skin leads to lipid peroxidation and reactive oxygen species causing skin lesions [7], the clinical features of which resemble sunburn [6]. These events show a tendency to occur early in the treatment and settle over time [5]. The management of the rash includes reduction of the drug dose and discontinuation of the drug in case of persistence of rash for more than 15 days. Slow re-introduction of the drug can be attempted once the symptoms have resolved [8]. It is critical both in initiating pirfenidone and in reintroducing it that the patients be educated of possible side-effects and photoprotection be strongly advised. Different studies on pirfenidone provide different statistics on the emergence of phototoxicity. Because it is still a rare phenomenon, more data are needed for the statistics to start converging. Till that time, it is important for the physicians to recommend necessary preventative actions thereby allowing the patient to continue benefitting from pirfenidone’s anti-IPF properties.

Even after the necessary preventative measures, the occurrence of phototoxicity, whether in the form of photosensitive or a photoallergic reaction can be treated with the use of oral and topical corticosteroids and antihistamines. Alongside the medication, avoiding direct sun exposure, wearing long-sleeved clothes, broad-brimmed hats, and use of broad-spectrum sunscreens with UVB and UVA protection also need to be practiced.

## Conclusions

Pirfenidone is a good example of how even with a drug that has a favorable benefit-risk profile, maintaining and following up on suspicions of side effects is important. Pirfenidone remains a novelty and widely beneficial treatment for IPF. Some patients, however, develop an eczematous rash due to the drug’s UV absorption properties. Keeping this in mind, respiratory physicians need to explain the importance of preventative photoprotective measures to all patients. The dermatologists who observe photosensitivity in patients must also carry forward the suspicion of the patient’s medicine/chemical usage.
